# Dysregulated brain regulatory T cells fail to control reactive gliosis following repeated antigen stimulation

**DOI:** 10.1016/j.isci.2023.106628

**Published:** 2023-04-08

**Authors:** Sujata Prasad, Amar Singh, Shuxian Hu, Wen S. Sheng, Priyanka Chauhan, James R. Lokensgard

**Affiliations:** 1 Neurovirology Laboratory, Department of Medicine, University of Minnesota, Minneapolis, MN 55455, USA; 2 Schulze Diabetes Institute Department of Surgery, University of Minnesota, Minneapolis, MN 55455, USA

**Keywords:** Components of the immune system, Immunology, Neuroscience

## Abstract

This study was undertaken to investigate the role of CD4+FoxP3+ regulatory T cells (Tregs) in regulating neuroinflammation during viral Ag-challenge and re-challenge. CD8^+^ lymphocytes persisting within tissues are designated tissue-resident memory T cells (T_RM_), within brain: bT_RM_. Reactivation of bT_RM_ with T cell epitope peptides generates rapid antiviral recall, but repeated stimulation leads to cumulative dysregulation of microglial activation, proliferation, and prolonged neurotoxic mediator production. Here, we show Tregs were recruited into murine brains following prime-CNS boost, but displayed altered phenotypes following repeated Ag-challenge. In response to repeated Ag, brain Tregs (bTregs) displayed inefficient immunosuppressive capacity, along with reduced expression of suppression of tumorigenicity 2 (ST2) and amphiregulin (Areg). *Ex vivo* Areg treatment revealed reduced production of neurotoxic mediators such as iNOS, IL-6, and IL-1β, and decreased microglial activation and proliferation. Taken together, these data indicate bTregs display an unstable phenotype and fail to control reactive gliosis in response to repeated Ag-challenge.

## Highlights

• Reduced ST2 and Areg expression by bTregs following Ag-re-challenge
• Areg suppresses microglial cell activation and production of neurotoxic mediators
• Brain Tregs show an unstable phenotype during repeated antigen exposure
• Reduced and dysfunctional Tregs display less capacity for immunosuppression

## Graphical abstract

**Figure:**
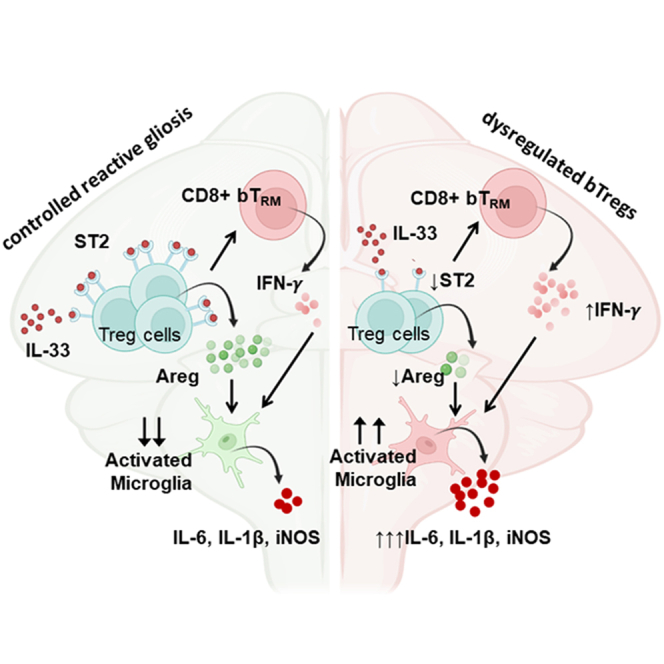

## Introduction

During inflammation, activated lymphocytes accumulate at sites of infection and damage. Effective immune responses play critical roles in clearing invading pathogens; however, prolonged or dysregulated immunity may result in exacerbated tissue destruction.^1^ Thus, sustained inflammatory responses may induce bystander damage to various tissues, particularly the brain, where uncontrolled inflammation causes severe injury to its generally non-regenerating cells.^2^ Therefore, the detrimental effects of these excessive neuroimmune responses need to be constrained.

There are relatively few T cells present in healthy brain. The lymphocytes that are present work in coordination with CNS-resident immune cells, to form the unique brain microenvironment needed for maintaining homeostasis. However, following viral brain infection, lymphocytes infiltrate to control the pathogen and restrict its spread. CD8^+^ T cells are key players in these adaptive immune responses. These CD8^+^ T cells perform the majority of antiviral defense of the brain through cytotoxic responses, as well as cytokine-mediated events. Furthermore, adaptive neuroimmune responses generate distinct subsets of memory CD8^+^ T cells which impart long-term protection. These long-term, persisting CD8^+^ lymphocytes have been designated tissue-resident memory T cells (T_RM_), within the brain: bT_RM_.^3^^,^^4^

Reactivation of T_RMs_ following stimulation with T cell epitope peptides generates strong and rapid antiviral recall responses.^3^^,^^5^^,^^6^ Persistent HIV-1 infection and reactivation from CNS reservoirs, even if intermittent, appears likely in cART-experienced patients. Immediate and effective pathogen control is mediated by abundant production of inflammatory cytokines and cytotoxic mediators; such as IFN-γ, TNF-α, perforin, and granzymes. The inflammatory cytokines produced by stimulated T_RM_ cells during an adaptive response can further activate resident innate immune cells leading to a tissue-wide antiviral state.^7^ In addition, T_RM_ cells trigger recruitment of additional circulating CD4^+^ T cells, CD8^+^ T cells, macrophages, B cells, and NK cells. Cytokine production, particularly that of IFN-γ, by bT_RM_ cells ensures a timely and robust effector response and inhibits viral spread; however, long-term pro-inflammatory cytokine production within brain is commonly associated with tissue damage and neuroinflammatory responses.

Using our well-established prime-CNS boost model, we have previously demonstrated that challenge with viral antigen (Ag) peptides, modeling viral reactivation events, amplified these adaptive immune responses. In addition, we have reported that repeated stimulation of adaptive immune responses from bT_RM_ leads to cumulative dysregulation of microglial cell activation, proliferation, and prolonged production of neurotoxic mediators.^8^ The heterologous prime-CNS boost/Ag-challenge and re-challenge model used here simulates cerebrospinal fluid viral escape, transient *de novo* viral Ag production, and its generation of subsequent adaptive, recall responses by brain-resident memory CD8(+) T cells. This murine model is useful in dissecting immune mechanisms which induce the neuroinflammation that drives bystander CNS injury. While virus replication-mediated tissue damage is a key feature during acute infection, these studies demonstrate that unchecked repeated or prolonged antiviral immune responses can also induce neuroimmunopathology.

Regulatory T cells (Tregs) are well known to play a major role in dampening excessive CD8^+^ T cell responses, preventing tissue damage and maintaining immune homeostasis. Given the potency of these cells in suppressing inflammation, the Treg subset is frequently studied in the context of autoimmune and degenerative diseases.^9^^,^^10^^,^^11^ Emerging evidence demonstrates that Treg cells not only maintain immune homeostasis in lymphoid tissue but they are also important in inhibiting responses in non-lymphoid tissues, such as the CNS,^8^^,^^12^^,^^13^ skin,^14^^,^^15^ and visceral adipose tissue.^16^^,^^17^ Studies in the context of brain disorders like ischemia, multiple sclerosis, Alzheimer's disease, CNS infection, and glioblastoma all show that T lymphocytes entering the brain contribute to inflammation-induced tissue injury.^18^^,^^19^^,^^20^^,^^21^ However, Treg cells infiltrate the CNS along with the other immune T cells. This infiltrating Treg population is indispensable in restraining aggressive neuroinflammatory responses. In addition, it has been recently reported that Treg cells within the brain are essential in suppressing astrogliosis and they perform specialized functions in neurological recovery.^8^ In addition, among patients with traumatic brain injury, the levels of circulating Treg cells predict their clinical outcome.^22^ However, despite their importance, little is known about the role of Tregs during repeated viral reactivation events such as those seen during chronic CNS infection.

The current study was undertaken to investigate the role of Treg cells in regulation of pathogenic T cell-induced neuroinflammation during viral Ag-challenge and re-challenge. Using our well-characterized heterologous prime boost model of HIV-associated neurocognitive disorder, we show that Treg cells are recruited into the murine brain following prime-CNS boost, but that their abundance is reduced following repeated Ag-challenge. Because of their inability to dampen inflammatory responses following subsequent reactivation events, we explored the phenotypic and functional roles of these brain Treg (i.e., bTreg) cells, particularly evaluating the role of amphiregulin (Areg). Areg is a growth factor known to have important roles in tissue repair and regeneration processes. Areg is widely produced by many epithelial and mesenchymal cells during the process of development and homeostasis. Areg mediates its biological activity via the epidermal growth factor receptor (EGFR). Treg-derived Areg has been found in various tissues such as lung and muscle, as well as brain, where it displays distinct roles in tissue repair. Here, we specifically investigated the role of Areg in modulation of neuroinflammatory responses.

## Results

### Decreased bTreg accumulation within the brain following repeated Ag stimulation

**Figure 1:**
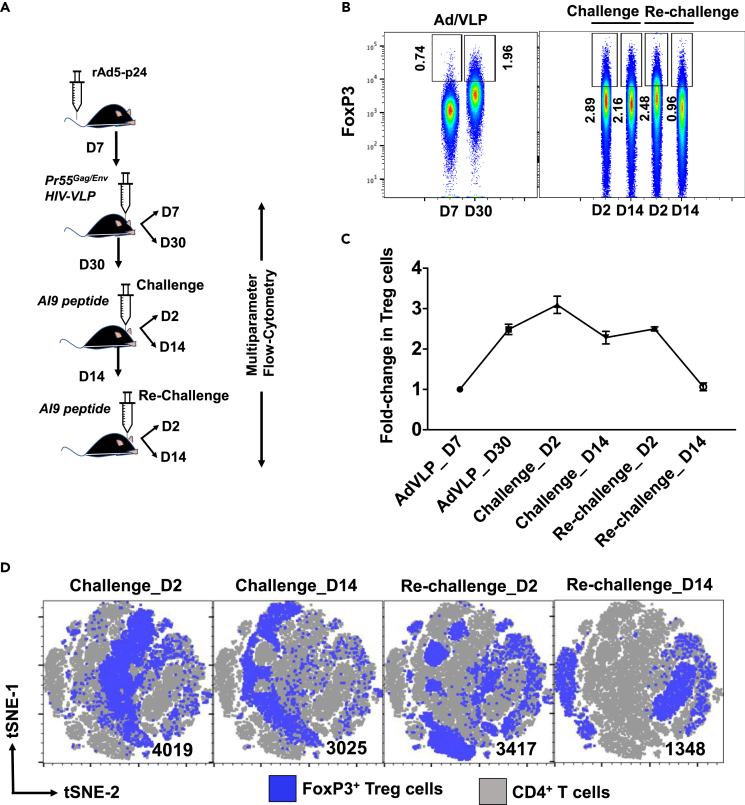
Decreased bTreg accumulation within the brain following repeated Ag-challenge (A) Schematic of the experimental design. Brain mononuclear cells (BMNC) were collected at days 7 and 30 post prime-CNS boost. To compare the impact of repeated Ag stimulation, BMNCs were also collected at days 2 and 14 post AI9 peptide challenge and re-challenge. (B) Pseudo color plots show the frequency of FoxP3-expressing CD4^+^ T cells analyzed on an equal cell number (132,000) generated from three individual concatenated FCS files (44,000 events/FCS) obtained from 6 animals per group per time points. (C) Kinetics of bTreg frequency at the indicated time points, data were analyzed using FlowJo plugins (v10.2). Data are expressed as mean ± SD. (D) tSNE analysis performed on concatenated FCS files show expression profiles of FoxP3 on CD4^+^ T cells at the indicated time points following AI9 peptide challenge and re-challenge.

We first evaluated the infiltration and persistence of Treg cells within murine brains following heterologous prime-CNS boost, as well as at various time points following Ag-challenge and re-challenge. Female BALB/c animals (6–8 weeks old) were primed by injecting a recombinant adenovirus vector expressing the HIV-1 p24 capsid protein (rAd5-p24) via the tail vein. Primed animals were then subjected to a CNS-boost using Pr55Gag/Env virus-like particles (HIV-VLPs) injected directly into the striatum. To evaluate the impact of Ag-challenge and re-challenge, prime-CNS boost animals were then subjected to *in vivo* Ag-challenge (day 30 post HIV-VLP injection) and subsequent re-challenge (day 14 post challenge) using the immunodominant AI9 CD8^+^ T cell epitope peptide (Figure 1A). Brain mononuclear cells (BMNCs) were isolated at days 7 and 30 post prime-CNS boost, as well as from animals subjected to Ag (AI9) challenge and re-challenge at days 2 and 14. We then analyzed the frequency of bTreg cells in the BMNCs using a FlowJo Plugin program. We observed that the frequency of CD4+FoxP3+ bTreg cells was initially low during the acute phase of infection (i.e., day 7 post prime-CNS boost), but this fraction increased within the brain by day 30 post prime-CNS boost (Figure 1B). We went on to examine CD4+FoxP3+ bTregs among groups which were subjected to Ag-challenge and re-challenge. In these studies, we observed that the bTreg fraction increased in number after Ag-challenge and re-challenge (i.e., at day 2), (Figures 1B and 1C). Among the animals that were subjected to Ag-challenge, a modest level of bTreg was maintained at day 14 post challenge; whereas, a dramatic reduction in the number of bTregs was observed among the re-challenged group at day 14 (Figures 1B and 1C). In addition, similar observations were made following analysis of bTreg cells by classical FlowJo analysis (Figures S1A and S1B). To assess the distribution of Tregs that were present within the brain following episodes of Ag-challenge and re-challenge, we visualized the Treg population using a tSNE dot plot. Analysis in this way uncovered a unique distribution pattern of bTreg cells at days 2 and 14 post re-challenge when compared to the challenged group. Most importantly, bTreg cells acquired unique locations on the tSNE dot plot at day 14 post re-challenge. These observations revealed that the bTreg population possessed a unique profile at day 14 following re-challenge (Figure 1D).

### Phenotypic characterization of bTregs reveals reduced expression of ST2 and Areg following Ag-re-challenge

**Figure 2:**
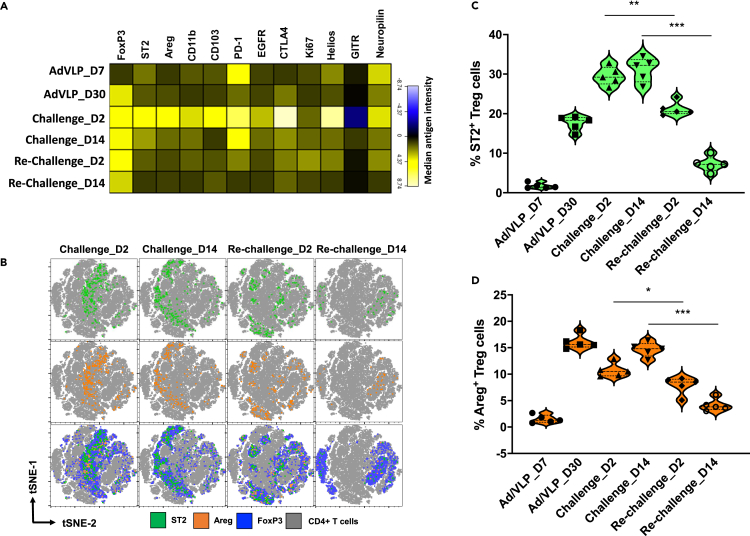
Phenotypic characterization of bTreg following AI9 challenge and re-challenge (A) Heatmap depicting differentially expressed markers on FoxP3+/CD4+ T cells at the indicated time points post prime-CNS boost, AI9 challenge, and re-challenge. (B) Two-dimensional tSNE analysis displaying ST2 expression (green color, top panel), and Areg production (orange color, middle panel) by bTreg (blue color, bottom panel) as well as their co-expression at different time points post AI9 challenge and re-challenge. Data were analyzed on equal cell numbers (132,000) generated from three individual concatenated FCS files (44,000 events/FCS) obtained from 6 animals per group per time point using FlowJo plugins (v10.2). (C) Violin plot showing the frequency of ST2-expressing FoxP3^+^ CD4^+^ T cells. (D) Violin plot showing the frequency of Areg-producing FoxP3^+^ CD4^+^ T cells. ∗p < 0.05*, ∗∗*p < 0.01*, ∗∗∗*p *<* 0.001.

We next examined bTreg phenotypic properties based on expression of 11 markers that define the activated state. Using flow cytometry and Cytobank, we analyzed cells obtained from prime-CNS boost animals, as well as from animals that were subjected to AI9-challenge and re-challenge at various time points. The flow-gating workflow, CD4^+^ T cell input, concatenation, and population clustering with tSNE algorithm are presented (Figures S2A–S2C). Heatmap data presented in (Figure 2A) visually present variations in expression of the 11 parameters between challenged and re-challenged groups. We also analyzed individual markers on equal cell numbers generated from individual concatenated FCS files from each time point following challenge and re-challenge (Figure S3A). Decreased expression of the bTreg cell markers: ST2, Areg, CD103, PD-1, EGFR, CTLA-4, Helios, GITR, and neuropilin were noted among the re-challenged group of animals both at days 2 and 14 when compared to the challenged group of animals (Figure 2A). The distribution pattern of ST2, Areg, and their co-expression on FoxP3+CD4^+^ bTregs was then assessed using comparative tSNE analysis of multi-color flow cytometry data (Figure 2B). We went on to further examine whether repeated restimulation events resulted in reduced bTreg ST2 and Areg expression and observed a rapid increase in the levels of both markers at day 2 post challenge. Furthermore, modest levels of ST2 and Areg expression were maintained until day 14 post challenge (Figures 2C and 2D). In sharp contrast, we observed a rapid decline in the levels of ST2 and Areg expression at both days 2 and 14 post repeated Ag-challenge (Figures 2C and 2D). The frequency of ST2 expression on bTregs was significantly lower in the AI9 re-challenged group (21.2% ± 1.7%) than the challenged group (29.5% ± 2.3%) at day 2 (Figure 2C). Similarly, ST2 expression was lower at day 14 post re-challenge (7.2% ± 1.9%), when compared to challenged group of animals (31.1% ± 3%) at day 14 (Figures 2C and 2D). Similar results were observed when analyzing the frequency of Areg-producing bTreg cells. Here, we found that Areg production was significantly higher among the challenged group of animals (10.7% ± 1.3%) when compared to the re-challenged group (7.9% ± 1.6%) at day 2. We also noted significantly lower levels of Areg expression at day 14 among the re-challenged group of animals (4.1% ± 1.1%) when compared to the challenged group (14.6% ± 1.3%), (Figures 2C and 2D). Concatenated FCS files also revealed decreased frequencies of ST2 and Areg expression on bTreg cells (Figure S3B).

### Amphiregulin treatment suppresses microglial cell activation, proliferation, and production of neurotoxic mediators

**Figure 3:**
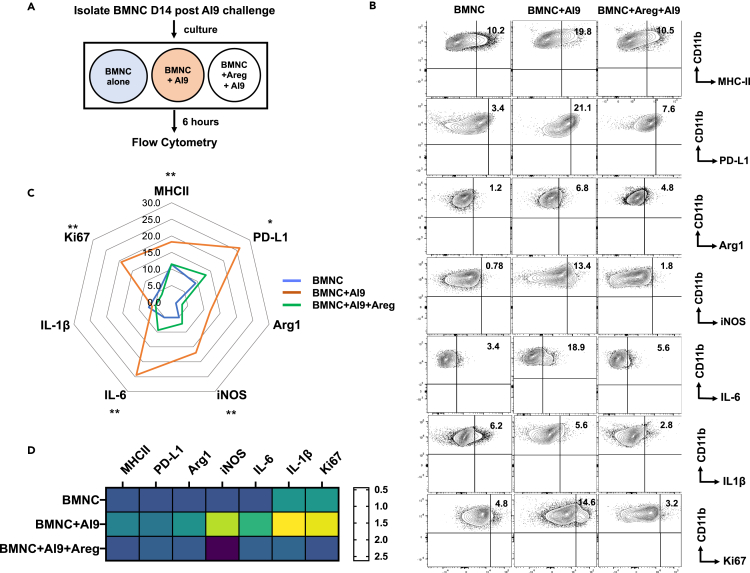
Amphiregulin suppresses neurotoxic marker production, activation, and proliferation of microglial cells (A) Schematic of the experimental design. BMNC were isolated from the AI9-challenged group of animals at day 14. Cells, with or without Areg treatment (2 h prior to AI9 re-challenge) were subjected to re-challenge with AI9 peptide *ex vivo* for 6 h. (B) Representative contour plots present expression of MHC-II, PD-L1, Arg1, iNOS, IL-6, IL-1B, and Ki67 on microglial cells (gated as CD45int/CD11b+/TMEM119+) under the indicated treatment. (C) Radar plot summarizing the mean frequencies of various makers expressed on microglia under the three conditions. (D) Heatmap analysis displaying the fold-change in median fluorescence intensity (MFI) of selected markers on microglial cells among different treatment groups. Normalized MFI value was used to calculate fold-change.

Having observed reduced expression of ST2 and Areg following Ag-re-challenge, we went on to specifically compare the involvement of Areg during these challenge and re-challenge events. We investigated the responses of BMNCs isolated from *in vivo* AI9-challenged animals (day 14) which were pretreated with or without recombinant Areg for 2 h. Following pretreatment, the BMNCs were then stimulated with AI9 peptide and flow cytometry was performed 6 h post *ex vivo* peptide re-challenge (Figure 3A). To assess the modulatory functions of Areg, we determined the expression profile of the microglial activation markers PD-L1 and MHC-II, evaluated expression of the proliferation marker Ki67, studied polarization using Arg1 expression, and investigated the production of neurotoxic mediators such as iNOS, IL-6, and IL-1β. As shown in Figures 3B and 3C, analysis revealed that microglia in BMNCs pretreated with Areg showed significantly reduced expression of MHC-II and PD-L1 when compared to untreated groups. Ki67 expression was also lower on microglia from the Areg-treated group, indicating a suppressed proliferative response. Interestingly, a significant reduction in IL-6 and iNOS expression was also observed among the Areg-treated cells. In addition, under untreated conditions microglia showed lower expression of Arg1, indicative of polarization into an inflammatory M1 phenotype. Furthermore, heatmap analysis of normalized expression for MHC-II, PD-L1, Arg1, iNOS, IL-6, IL1β, and Ki67 under the indicated treatment conditions also shows that Areg treatment suppressed neuroinflammatory responses (Figure 3D).

### Amphiregulin promotes anti-neuroinflammation in peptide-stimulated BMNC cultures through regulating IFN-γ production

**Figure 4:**
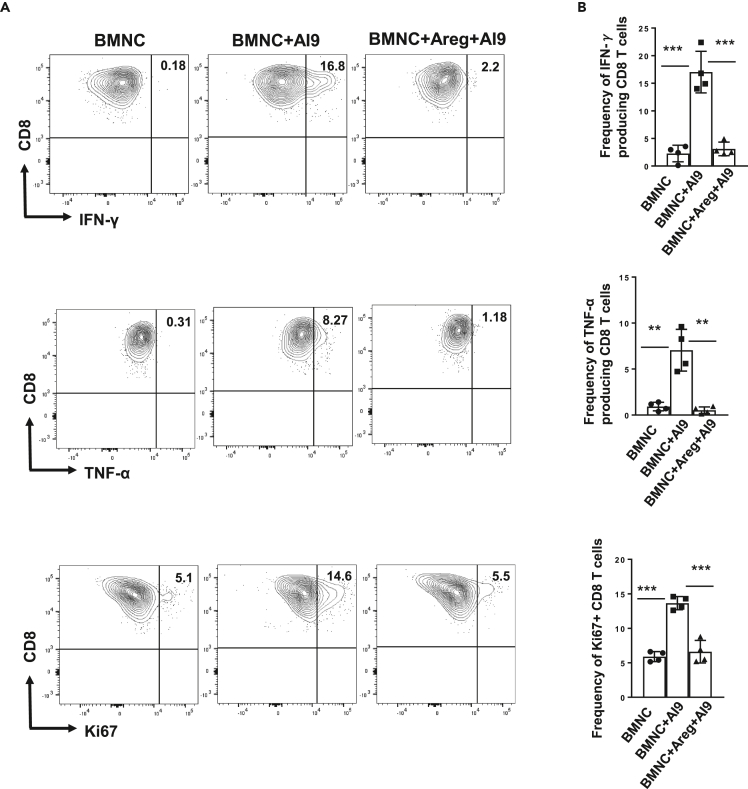
Amphiregulin suppresses inflammation by regulating IFN-γ production (A) Representative contour plots display production of the pro-inflammatory cytokines IFN-γ and TNF-α, as well as Ki67 expression profile on CD8^+^ T cells under different treatment conditions. (B) Bar graphs show frequencies of IFN-γ- and TNF-α-producing, as well as proliferating CD8^+^ T cells under the indicated treatment conditions. Results shown are from data obtained in two independent experiments. *∗∗*p < 0.01*, ∗∗∗*p *<* 0.001.

We next examined the role of Areg in modulating inflammatory responses from peptide-stimulated CD8^+^ T cells. In these experiments, BMNCs isolated from *in vivo* AI9-challenged animals (d 14) with and without Areg treatment were stimulated with AI9 peptide, 2 h post exposure to Areg. We subsequently assessed the production of inflammatory cytokines, as well as the proliferative response of CD8^+^ T cells. Interestingly, we observed significantly reduced production of pro-inflammatory cytokines, such as IFN-γ and TNF-α, among the Areg-treated group when compared with BMNCs that were stimulated with AI9 peptide without Areg treatment (Figures 4A and 4B). In addition, when evaluating the proliferative responses of CD8^+^ T cells with and without Areg treatment, we observed significantly reduced expression of IFN-γ, TNF-α, and Ki67 (Figures 4A and 4B). In sharp contrast, CD8^+^ T cells showed increased expression of Ki67 when these cells were stimulated with AI9 peptide in the absence of Areg treatment (Figures 4A and 4B).

## Discussion

Treg cells limit tissue injury and promote repair process, thereby maintaining homeostasis. The association between Tregs and repair is evident in various tissues such as kidney, liver, and lung, as well as brain.^23^^,^^24^ It has been observed that Treg cells infiltrate inflamed tissues and modulate production of pro-inflammatory cytokines by other immune cells. Similarly, our current findings demonstrate that Tregs infiltrated the brain of heterologous prime-CNS boost animals and modulated neuroinflammatory responses during subsequent Ag-challenge. In addition, *in vitro* studies showed a similar association between Treg cells and tissue repair process. These pro-repair and pro-regenerative roles for Tregs both during acute and chronic phases of infection have been studied for decades. However, studies indicate Treg dysfunction, which fails to suppress effector T cell responses, may also occur during chronic infection.^25^^,^^26^^,^^27^ The findings reported here, in which multi-color flow analysis of brain tissue revealed that the number of Treg cells decreased following subsequent Ag-re-challenge events, are in line with these previous studies.

Tregs undergo phenotypic and functional changes in response to inflammatory challenges. We have previously reported that inappropriately prolonged neuroinflammatory responses were observed following restimulation with T cell epitope peptides, due to elevated levels of pro-inflammatory cytokines produced by activated bT_RMs_.^28^ In this study, we investigated the functional role of Tregs in controlling neuroinflammatory responses during repeated Ag-challenge and re-challenge events. We found that the numbers of FoxP3-expressing CD4^+^ T cells decreased within the brain following prolonged neuroinflammatory responses. Changes in the location of FoxP3+ cells at days 2 or14 post AI9 re-challenge on tSNE map indicate that they are distinct and discrete from the challenge group, which is likely due to new Treg cells developing in brain. We also observed that these Tregs lost phenotypic and functional markers like Areg and ST2, rendering them dysfunctional and positioning them to different continent of the tSNE map, distinct from Areg- and ST2-expressing cells. Our observations were contrary to peripheral counter parts, where no significant alteration in the relative number of Treg cells was noted among patients with myasthenia gravis.^28^ These findings indicate that Treg number is influenced by development, persistence, and proliferation within a particular tissue microenvironment. Besides the decrease in FoxP3 expression, our tSNE analysis revealed unique distribution patterns of bTregs at days 2 and 14 post re-challenge. These data suggest that bTregs display different profiles during events of repeated Ag-challenge.

Accumulating evidence suggests that Treg cells from an inflamed environment may lose FoxP3 expression, leading to an unstable phenotype which ablates their suppressive function.^29^^,^^30^^,^^31^ Treg cells which lose FoxP3 expression are classified as ex-Tregs.^32^ Ex-Tregs do not show suppressive function and are considered to be key players in autoimmune responses.^32^ Repetitive *in vitro* stimulation has also been demonstrated to result in loss of FoxP3 expression on Tregs.^33^ Under these conditions, Treg cells readily convert into diverse effector cell types, impairing immune homeostasis and aggravating immune-mediated disorders.^34^^,^^35^^,^^36^ Several studies suggest that pro-inflammatory cytokines, particularly IL-6 and IL-1β, reduce Treg stability, thereby promoting their conversion to a Th17 phenotype.^37^^,^^38^^,^^39^

In the present study, phenotypic profiling of Tregs from Ag-challenge and re-challenged animals revealed phenotypic diversity. bTregs expressed high levels of CD103, PD-1, EGFR, CTLA-4, Helios, GITR, ST-2, Areg, Ki67, and neuropilin following infection. However, a decrease in the expression of these parameters was observed among the re-challenged group. These data indicate that bTregs display an unstable phenotype during repeated Ag exposure. Similarly, other studies have also demonstrated that the Treg subset might not be able to undergo effective recall expansion following Ag stimulation.^40^ Like Tregs in muscle and visceral adipose tissue, bTregs respond to IL-33 and express high levels of the IL-33 receptor ST2.^8^ It has been reported that IL-33 is constitutively produced within the brain and its level increases during infection and injury.^8^ bTregs exhibited an enhanced suppressive phenotype following Ag-challenge. However, this suppressive activity was disrupted following repetitive Ag stimulation. Alterations in the phenotypic profile of Treg cells were noted in the presence and absence of repeated activation signals. Reduced levels of activated cell markers along with reduced expression of Areg and ST2 among re-challenged animals indicated that Tregs are altered in their phenotype during repeated inflammatory events. Moreover, reports from other studies show reduced Treg cell number within the injured brain of ST2-deficient mice.^8^ In addition, as expected, *ex vivo* Areg treatment revealed reduced production of neurotoxic mediators such as iNOS, IL-6, and IL-1β as well as decreased microglial activation and proliferation. Similarly, other studies report that Treg cells from Areg-deficient mice fail to suppress astrogliosis.^8^ Moreover, it has been reported that microglial cells and astrocytes from injured brains of Treg cell-depleted mice express increased levels of IL-6.^8^ Areg-producing Treg cells specifically downregulate IL-6 and depletion of these cells activates IL-6 and STAT-3 signaling pathways.^8^ These data indicate that reduced ST2 and Areg production during repeated reactivation events failed to suppress prolonged neuroinflammatory responses and promote tissue repair.

Failure to control prolonged adaptive immune responses is believed to augment exuberant, overzealous neuroinflammation. Treg cells protect from these overwhelming responses, but simultaneously restrict CD8^+^ T cells from effectively performing their function. Previous studies have demonstrated that Tregs effectively suppress both the proliferation and IFN-γ secretion by virus-specific CD8^+^ T cells in patients infected with hepatitis C virus.^41^ Areg production has been reported to enhance the immunosuppressive properties of hepatic Tregs.^42^ Similarly, in this study, we noted that Areg reduced inflammatory cytokine production, particularly that of IFN-γ by CD8^+^ T cells, during repeated Ag-challenge. In addition, our results also demonstrate that treatment with Areg effectively suppressed CD8^+^ T cell proliferation. Taken together, these data indicate that ST2 expression and Areg production modulate neuroinflammatory responses during Ag restimulation and may lead to new therapeutic strategies for control of degenerative damage induced by repeated neuroinflammation.

### Limitations of the study

It is currently unknown why HIV neurocognitive impairment persists despite effective viral suppression to undetectable levels in most cART-treated individuals. It is also unclear whether the impairment is instigated by aberrant immune responses driven by repeated Ag exposure, as modeled here. Viral escape within the brain, and its generation of subsequent adaptive, recall responses by brain-resident CD8+ T cells to control spread, may induce neuroinflammation that drives bystander CNS injury, but the development of neurocognitive impairment likely involves additional mechanisms. While no animal model for any disease is perfect, they are frequently utilized and essential for preclinical testing of innovative treatments. Clearly, our heterologous prime-CNS boost model along with direct Ag injection into the brain cannot exactly reflect the outcome of viral reactivation events and their associated *de novo* production of viral Ag. These caveats regarding our model’s translational relevance are acknowledged. Further studies investigating immune responses to persistent viral brain infections using reactivate-able live virus models will help dissect the contributions of neuroimmunopathology versus those of direct viral replication.

## STAR★Methods

### Key resources table

**Table:** 

REAGENT or RESOURCE	SOURCE	IDENTIFIER
**Antibodies**		

anti-PD-L1-BV605	Biolegend	Catalog # 153606
anti-CD11b-AF700	Thermo Fisher Scientific	Catalog # 56-0112-82
anti-Arginase1-FITC	Biotechne (R&D)	Catalog #IC5868SF
anti-CD8-eF450	Thermo Fisher Scientific	Catalog # 48-0081-82
anti-Amphiregulin-biotinylated	Biotechne (R&D)	Catalog # BAF 989
Steptavidin-APC	Biotechne (R&D)	Catalog #F0050
anti-EGFR-FITC	Novo Biologicals	Catalog # NB-600-724F
anti-ST2-PerCP-eF710	Thermo Fisher Scientific	Catalog # 46-9333-80
anti-CTLA-4-PE-CF594	BD Bioscience	Catalog # 564332
anti-GITR-APC-eFluor780	Thermo Fisher Scientific	Catalog # 47-5874-82
anti-MHCII-BV510	Biolegend	Catalog # 107636
anti-TMEM119-PerCP-eF710	Thermo Fisher Scientific	Catalog # 46-6119-82
anti-CD45-BV785	Biolegend	Catalog # 103149
anti-PD-1-BV711	Biolegend	Catalog # 135231
anti-CD4-BV510	Biolegend	Catalog # 116025
anti-CD103-BV605	Biolegend	Catalog # 121433
anti-IL-1β- APC-eF780	Thermo Fisher Scientific	Catalog # 47-7114-82
anti-FoxP3-PE	Thermo Fisher Scientific	Catalog # 12-5773-82
anti-IL-6-eF450	Thermo Fisher Scientific	Catalog # 48-7061-82
anti-iNOS-PE	Thermo Fisher Scientific	Catalog # 12-5920-82
anti-Helios-eF450	Thermo Fisher Scientific	Catalog # 48-9883-41
anti-Ki67-PECy7	Thermo Fisher Scientific	Catalog # 25-5698-82
anti-neuropilin-BV650	BD Bioscience	Catalog # 752455
anti-IFN-γ-APC	Thermo Fisher Scientific	Catalog # 17-7311-82
anti-TNF-α-BV711	Biolegend	Catalog # 506349

**Bacterial and virus strains**		

rAd5-p24	VectorBuilder Inc	N/A

**Chemicals, peptides, and recombinant proteins**		

AI-9 (MHC class I-restricted peptide AMQMLKETI)	MBL International Corp	N/A

**Experimental models: Organisms/strains**		

BALB/c	Charles River Laboratories	N/A

**Software and algorithms**		

Flowjo Plugin software	FlowJo, Ashland, OR	https://www.flowjo.com/solutions/flowjo/downloads

### Resource availability

#### Lead contact

Any further information and request related to reagents or resource sharing should be directed to and will be fulfilled by lead contact, James R. Lokensgard, e-mail: (loken006@umn.edu).

#### Materials availability

This study did not generate new unique reagents.

### Method details

#### Experimental models

Six-to eight-week-old, female BALB/c mice (Charles River Laboratories International, Wilmington, MA) were used for this study. All animal experiments were pre-approved by the University of Minnesota Institutional Animal Care and Use Committee. This study was performed in strict accordance with recommendations in the Guide for the Care and Use of Laboratory Animals of the National Institutes of Health. Surgery was performed under Ketamin/Xylazine anesthesia, and all efforts were made to minimize suffering. Following surgery, all animals were routinely cared for in accordance with RAR (Research Animal Resources) procedures before being euthanized using isoflurane. Institutional Biosafety Committee (IBC) guidelines and recommended procedures were strictly followed.

#### Mice maintenance/diet

Mice were maintained in a temperature-controlled facility with a 12-h light/dark cycle with free access to food and water. The standard chow diet was used for regular maintenance and breeding. Mice were giver soft chow following surgery.

#### Virus and animals

We used our previously described heterologous prime-boost model. Production of a second-generation (i.e., ΔE1 + ΔE3), replication-incompetent adenovirus vector which expresses the HIV-1 capsid protein p24 under control of the minimal CMV IE promoter (i.e., rAd5-p24) was outsourced to VectorBuilder Inc. (Chicago,IL). Six-to eight-week-old, female BALB/c mice were infected with rAd5-p24 (1 x 10^10^ PFU/mouse) through tail-vein injection. Animals were then boosted (d7 later) via an intracranial injection of HIV virus-like particles (HIV-VLPs). To promote immune cell infiltration and retention within the brain, animals were injected with 300 fluorescent units (FU) of HIV-VLP into the striatum via stereotaxic injection in a volume no greater than 5 μL.

#### Production of HIV virus-like particles (HIV-VLPs)

Production of HIV-VLPs was performed by transfection of HEK 293T cells with pEYFP-N3 HIV-1, a Gag-expressing codon-optimized plasmid encoding the 55 kDa Gag precursor protein fused to enhanced yellow fluorescent protein (EYFP), under control of the human CMV IE promoter, obtained from Louis Mansky (Institute for Molecular Virology, University of Minnesota); p3NL(ADA) env, encoding a full length R5-tropic envelope protein, under control of the HIV-1 LTR promoter, was obtained with permission from Eric Freed (NCI, Frederick, MD). Because these HIV-VLPs express EYFP, fluorescent units (FU) were used as an indicator of quantity and were quantified using a Spectramax M2 Fluorescent reader [485/538 nm (ex/em) 530 nm cutoff]. An HIV-VLP dose of 300 FU equivalents conferred reliable stimulation and promoted peripheral immune cell infiltration into the brain.

#### Intracranial injection of mice

For the precise injection of HIV-VLPs or AI9 peptide into the striatum, intracranial injection of mice was performed as previously described with slight modifications.^24^ Briefly, injection into the 6–8-week-old BALB/c female animals was performed by anesthetizing animals using an appropriate dose of Ketamine and Xylazine (100 mg and 10 mg/kg body weight, respectively). The mixture was injected intraperitoneally. After checking for the lack of response by toe pinch, animals were immobilized on a small animal stereotactic instrument equipped with a Cunningham mouse adapter (Stoelting Co., Wood Dale, IL). To minimize pain, prior to incision, subcutaneous injection of the analgesic bupivacaine (1–2 mg/kg (0.4–0.8 ml/kg of a 0.25% solution), Hospira, Inc. Lake Forest, IL) was given. Straight midline incision was made, and connective tissues were softly pulled aside to expose reference sutures (sagittal and coronal) on the skull. The sagittal plane was adjusted in a manner that bregma and lambda were positioned at the same coordinates on the vertical plane. A single burr hole was drilled to expose the underlying dura at pre-determined coordinates to access the left striatum (AP = 0 mm, ML = 2.0 mm from bregma, and DV = 3.0 from skull surface). The exposed skull was kept moist using sterile saline. Animals received 300 FU of HIV-VLP in a volume no greater than 5 μl delivered to the striatum via stereotaxic injection, using a Hamilton syringe (10 μl) fitted to a 27 G needle. For proper delivery of the injection and to avoid, acute increase in intracranial pressure, injection was delivered slowly 1 μl/min over a period of 5 min. To avoid back flow of fluid, the needle was retracted slowly, and the burr hole was closed using sterile bone-wax. The animals were then removed from the stereotaxic apparatus, placed on heating pad and the skin incision was closed with 4-0 silk sutures with a FS-2 needle (Ethicon, Somerville NJ).

#### Ag challenge and re-challenge

The capsid p24 H-2K^d^ MHC class I-restricted peptide AMQMLKETI (i.e., AI9) has been identified as an immunodominant epitope.^25^^,^^26^ To determine the impact of repeated Ag stimulation we injected animals with AI9 peptide (MBL International Corp., Woburn, MA). 30 days post-HIV-VLP administration, animals were subjected to *in vivo* challenge (i.e., first AI9 injection) whereas re-challenge (i.e., second AI9 injection) was performed at d 14 post-challenge. Animals were injected with 100 μM AI9 peptide in 5 μl saline delivered into the striatum. Brain tissue was isolated at d 2 and 14 post-challenge and re-challenge. Freshly isolated brain derived mononuclear cells (BMNC) were examined by flow cytometry.

#### Brain leukocyte isolation and flow cytometry analysis

BMNCs were isolated from brains of prime-CNS boost, Ag-challenged and re-challenged groups of animals using a previously described procedure with minor modifications.^24^^,^^27^^,^^28^^,^^29^ Briefly, whole brain tissues were harvested (n = 6 animals/group/experiment), minced finely using a scalpel, and then stirred on a magnetic stirrer for 30 min in RPMI 1640 (2 g/L D-glucose and 10 mM HEPES) without any enzymatic treatment. Single cells prepared were resuspended in 30% Percoll (Sigma-Aldrich, St. Louis, MO) and banded on a 70% Percoll cushion at 900 × g for 30 min at 15°C. Brain leukocytes were collected from the 30–70% Percoll interface and treated with Fc block (anti-CD32/CD16 in the form of 2.4G2 hybridoma culture supernatant with 2% normal rat and 2% normal mouse serum) to inhibit non-specific Ab binding. Cells were then counted using the trypan blue dye exclusion method, and 1 x 10^6^ cells were subsequently stained using anti-mouse immune cell surface markers for 15–20 min at 4°C in the dark. The surface markers used were anti-CD11b-AF700, anti-CD8-eF450, anti-ST2-PerCP-eF710, anti-GITR-eFluor 780, anti-MHCII-APC, anti-TMEM119-PE (Thermo Fisher Scientific, Waltham MA), anti-CTLA-4-PE-CF594 (BD Bioscience), anti-EGFR-FITC (Novo Biologicals, Biotechne brand), anti-CD103-BV605, anti-CD45-BV785, anti-PD-1-BV711, anti-CD4-BV510, and anti-PD-L1-BV605 from (Biolegend, San Diego, CA). The cells were then washed using FACS buffer and fixed using fixation buffer for 20 min at RT. Control isotype Abs were used for all fluorochrome combinations to assess nonspecific Ab binding. Samples were acquired using an LSRFortessa-X20 (BD Biosciences, San Jose CA) flow cytometer. Live leukocytes were gated using forward scatter and side scatter parameters and data were analyzed using FlowJo software (FlowJo, Ashland, OR).

#### Intracellular staining

Intracellular staining was performed to assess expression of FoxP3, as well as markers associated with the activated state of Treg cells (anti-Helios, anti-neuropilin-1, a transmembrane glycoprotein, and anti-Areg). To assess production of proinflammatory and anti-inflammatory mediators; and the proliferative response of microglia, intracellular staining was performed. We evaluated the expression of interleukin (IL)-6, IL-1β, iNOS (inducible nitric oxide synthase), Arg1 (Arginase1), Areg, and Ki67. BMNC (2 x 10^6^ cells/well) were surface stained prior to fixation/permeabilization using cytofix/cytosperm kit (eBioscience now Thermo Fisher Scientific). Cells were then stained using anti-FoxP3-PE, anti-IL-6-PerCP-eFluor450, anti-iNOS-PE, anti-Helios-eF450, and anti-Ki67-PECy7 (Thermo Fisher Scientific), anti-IL-1β- BV711 (Biolegend, San Diego, CA), Arg1-FITC, anti-Areg-biotinylated-streptavidin-APC (R&D Systems, Minneapolis, MN) and anti-neuropilin-BV650 (BD Bioscience) as recommended by the manufacturer’s protocols. Stained and fixed cells were analyzed using flow cytometry as described above.

#### *Ex vivo* AI9 re-challenge

Female BALB/c animals (6–8 wk), primed by injecting recombinant adenovirus vectors expressing HIV-1 p24 capsid protein (rAd5-p24) via tail vein injection were then subjected to CNS-boost using Pr55Gag/Env virus-like particles (HIV-VLPs), injected with AI9 into the striatum as described above. BMNC were collected from these animals at d 14 post *in vivo* Ag-challenge. These cells were then pretreated with and without recombinant mouse Areg (R&D Systems) for 2h and then stimulated with AI9 peptide *ex vivo*. Flow cytometry was performed 6h post *ex vivo* peptide re-challenge.

#### tSNE analysis

For tSNE analysis, total CD4^+^ T-cells were selected from pre-gated total CD45hi leukocytes. Samples were downsized to derived FCS files with an equal number of CD4^+^ T-cells from each time point D2 and D14 post AI9 challenge, as well as D2 and D14 post AI9 re-challenge. Later these files were concatenated using FlowJo software and single FCS files were generated for subsequent tSNE clustering analysis. The tSNE map of total CD4^+^ T-cells was built on key markers (FoxP3, ST2, Areg, CD11b, CD103, PD-1, EGFR, CTLA-4, Ki67, Helios, GITR and Neuropilin).

### Quantification and statistical analysis

Statistical analyses were performed using GraphPad prism 7.03 software, San Diego. Significant differences were calculated based on Student’s T-test. A value of p < 0.05 was considered significant.

## Data Availability

• Data reported in this paper will be shared by the lead contact upon request. • No sequence data was generated for this study. • This paper does not report original code. • Any additional information required to reanalyze the data reported in this paper is available from the lead contact upon request.
