# Supplementary material for: Dysregulated brain regulatory T cells fail to control reactive gliosis following repeated antigen stimulation
Source: iScience. 2023 Apr 8;26(5):106628. doi: 10.1016/j.isci.2023.106628 (PMC10182273; doi:10.1016/j.isci.2023.106628)
Supplement: Document S1. Figures S1–S3 [file mmc1.pdf]

iScience, Volume *26*

Supplemental information

Dysregulated brain regulatory T cells fail to control reactive gliosis following repeated antigen stimulation

Sujata Prasad, Amar Singh, Shuxian Hu, Wen S. Sheng, Priyanka Chauhan, and James R. Lokensgard

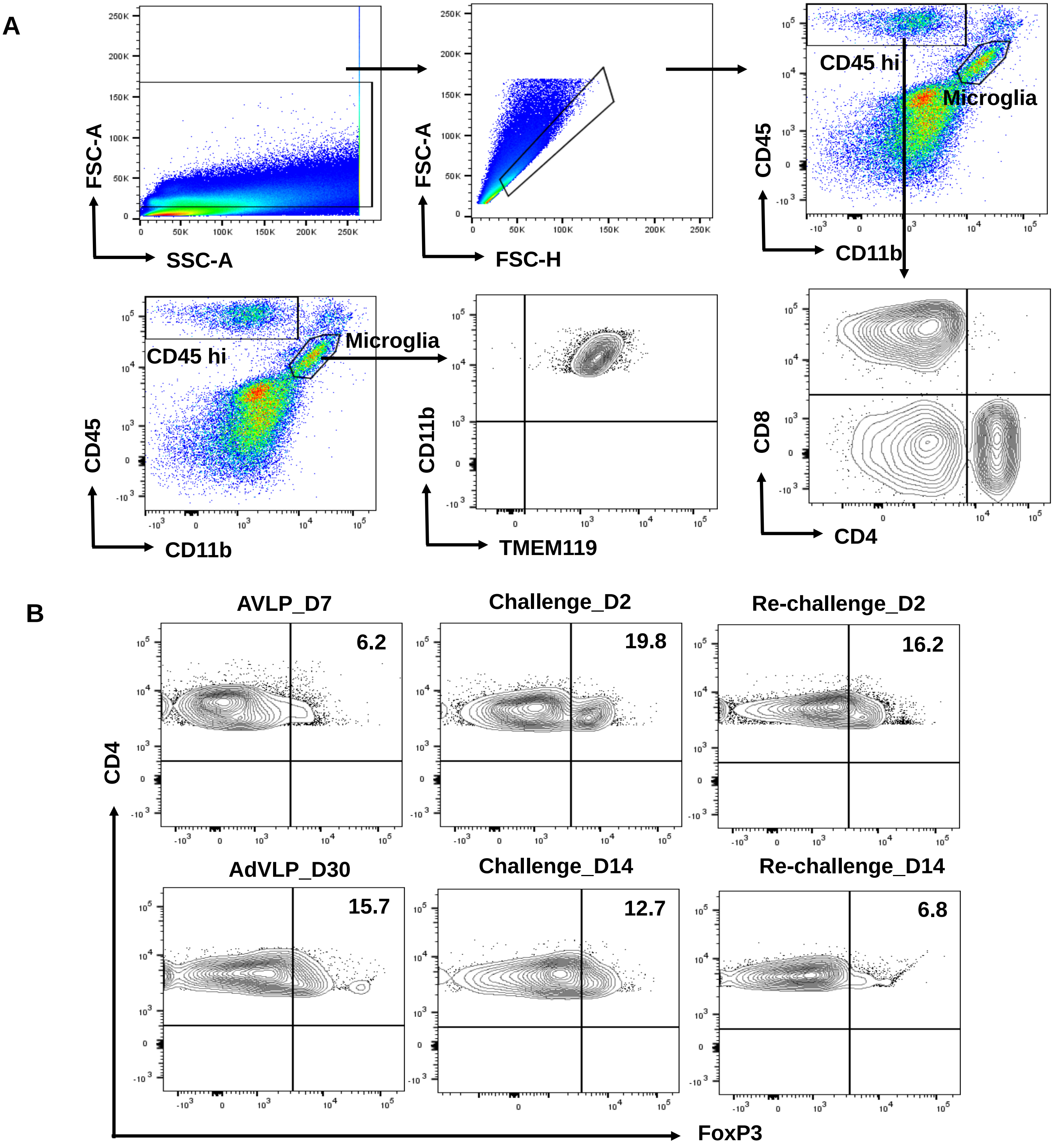

Figure S1. Flow Gating Strategy. Related to Figure 1

A. Gating strategy to identify major immune cell populations within the brain. Microglial cells were identified as the CD45int/CD11+ population, as well as by co-expression of

CD11b and TMEM119. CD4+ and CD8+ T-cells were identified within the gated CD45hi population **B.** Representative contour plot show expression of FoxP3 on CD4+ T-cells following prime-CNS-boost, AI9 peptide challenge, and re-challenge at the indicated time points.

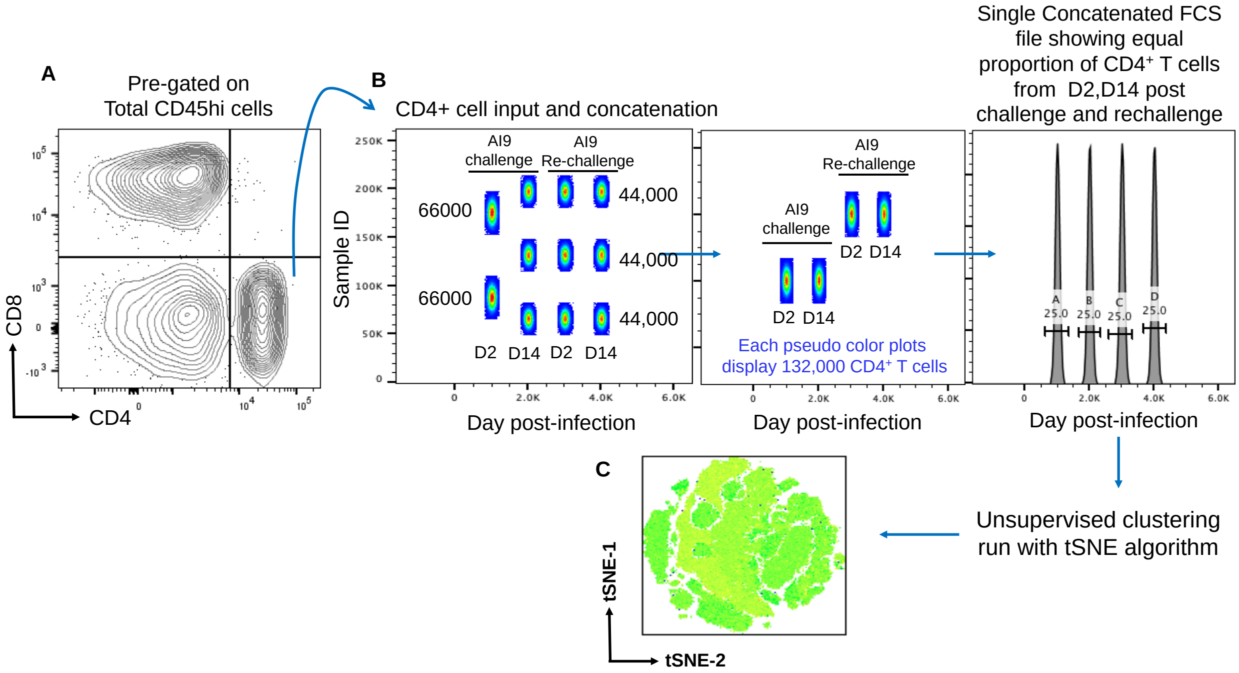

Figure S2. Flow-gating workflow, CD4+ T cell input, concatenation and population clustering with the tSNE algorithm. Related to Figure 2.

A. Total CD4+ T-cells were selected from pre-gated total CD45hi leukocytes. **B.** Samples were downsized to derived FCS files with equal number CD4+ T-cells from each time points D2 and D14 post AI9 challenge and D2, D14 post AI9 re-challenge. Later these files were concatenated using FlowJo software and single FCS files were generated for subsequent tSNE clustering analysis. tSNE map of total CD4+ T-cells was built on key markers (ST2, Areg, CD11b, CD103, PD-1, EGFR, CTLA-4, Ki67, Helios, GITR and Neuropilin). **C.** tSNE map of concatenated cells showing various immune

clusters of CD4+ T-cells grouped into distinct continent on the two-dimensional tSNE map.

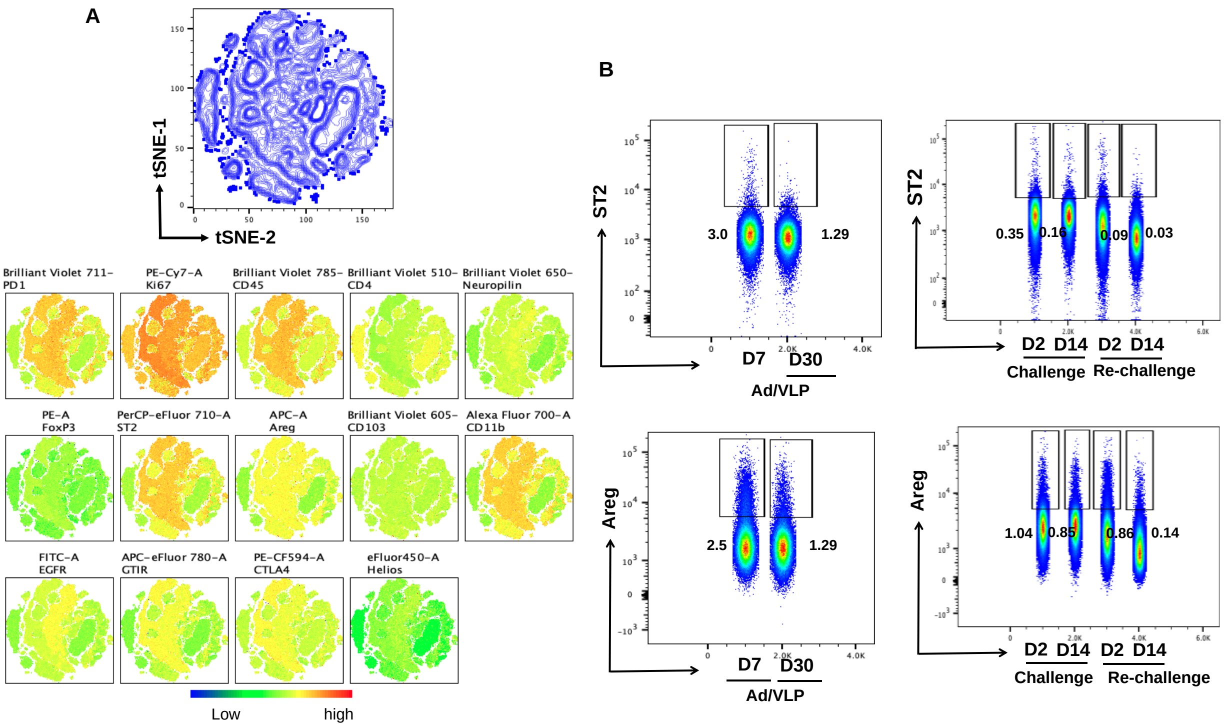

Figure S3: Phenotypic expression of each individual marker. Related to Figure 2. **A.** Two dimensional tSNE plots showing the expression of individual markers analyzed on equal cell numbers generated from individual concatenated FCS files from all time points following challenge and re-challenge. **B.** Pseudocolor FACS plots show the expression of ST2 (Upper panel) and Areg (Lower panel) on CD4+ T cells at d 7 and d 30 before challenge, as well as d 2 and d 14 post challenge and re-challenge.
